# The effects of malapportionment on economic development

**DOI:** 10.1371/journal.pone.0259150

**Published:** 2021-12-01

**Authors:** Rikhil R. Bhavnani

**Affiliations:** Department of Political Science, University of Wisconsin–Madison, Madison, WI, United States of America; Sam Houston State University, UNITED STATES

## Abstract

Does the unequal formal representation of people in legislatures (“malapportionment”) affect development? Answering this question is critical for assessing the welfare costs of malapportionment. We argue that representation might spur development as the desire for reelection incentivizes legislators to provide for their districts, and as voters hold politicians to account for doing so. Since this is the case, malapportionment might cause unequal development. Using data from India, we show that a 10% increase in representation causes a 0.6% increase in night lights, a frequently used proxy for development. Reapportionment, or the equalization for representation, attenuates this effect. Consistent with the theory, the effect of representation is larger in districts with legislators and voters that are able to hold the executive to account.

## 1 Introduction

The empirical case against unequal legislative representation or malapportionment is incomplete. Although a voluminous literature examines the effects of unequal representation on coalition composition [1, 2], the distribution of funds [3–5] and public policy [6], we know of no work that examines its effects on economic development. Yet, understanding whether and why malapportionment affects development—which is arguably the ultimate outcome of the other outcomes studied in the literature—is critical for assessing the welfare costs of unequal representation.

We draw on the usually separate literatures on malapportionment reviewed above and the distributive politics literature [7] to argue that unequal legislative representation might affect developmental outcomes. We focus on two of the many reasons that this could be the case. First, politicians might wish to improve the development outcomes of their constituencies to be reelected. And second, voters might work to ensure that politicians are held accountable for the delivery of better development. As we argue below, the operation of both mechanisms is increasing in the relative representation of districts. Since representation affects development, malapportionment or unequal representation causes unequal development.

To test our argument, we examine the effects of malapportionment on night light output across India’s administrative districts. A substantial literature employs night lights as a measure of development outcomes, particularly in contexts where development is hard to track [8–10]. India is an appropriate case because although the degree of malapportionment is around the world average [11], the country has substantial sub-national variation in the degree of unequal political representation [1].

Our analysis suggests that a 10% increase in representation increases light output by 0.6%, which is the equivalent of 25% of the average annual increase in light output. We also find that reapportionment attenuates the effect of malapportionment. Our results are robust to examining the effect of malapportionment on another development outcome and a number of specification changes. Lastly, the data suggest that the benefits of increased representation are concentrated, as suggested by the theory, in places with powerful legislators and informed electorates.

In addition to contributing to the literature on malapportionment, our paper contributes to the literature on democracy and development. Much of this literature focuses on the “extensive” margin, examining the effects of transitions to democracy on development [12]. In this paper, we focus on the “intensive” margin, examining whether more or more equal representation within a large democracy improves development. In this sense, our paper is similar to the burgeoning literature on the effects of the deepening of the franchise [13] on development.

## 2 How malapportionment affects development

Building on the literature, we focus on bottom-up and top-down reasons that representation could affect development. Since representation affects development, unequal representation or malapportionment leads to unequal development.

Note that at the level of the district—which is the main administrative unit in India, and is the unit of analysis for the study—reapportionment increases, decreases or leaves unchanged the number of single-member constituencies and therefore the number of politicians that represent each district. I argue that reapportionment improves development if it gives underrepresented districts more politicians, particularly if these politicians are powerful (that is, in the governing coalition) and/or if voters are able to hold them to account.

First, to take a bottom-up view, recall that development occurs as voters hold politicians to account. For example, research shows that India’s central government is more likely to respond to food shortfalls in states with more informed electorates [14]. If reapportionment grants a district more single-member constituencies, the average constituency is smaller, and politicians become more accessible and accountable to voters. This improves development. In larger constituencies, voter-politician interactions are less likely to occur and are more likely to be mediated by interest groups. This impedes accountability and development. While the opposite is possible—more representatives could make it difficult for voters to figure out who to approach, or how to apportion credit or blame, and might therefore undermine accountability [15]—this possibility is attenuated by the fact that India’s districts have relatively few (an average of seven) single-member districts, which helps voters to connect outcomes with politicians [16]. We also draw on the literature to note that we expect that certain types of voters—such as those that are informed—might especially be able to hold politicians to account [14, 17].

A second reason that development might be increasing in representation is top-down. Politicians frequently try to secure their reelection by targeting particular constituencies for benefits that, in turn, might impact development [18, 19]. In the context of India, politicians boost the supply of electricity to constituencies aligned with the ruling party or coalition [20], particularly around elections [21]. National politicians also channel resources to places with copartisan state legislators [22]. Although this literature identifies different logics for the distribution of government resources (for example, there is disagreement over whether core or swing voters or districts are targeted), all these mechanisms could conceivably be increasing in the number of representatives per capita (or decreasing in malapportionment), as more legislators can exert more effort to improve development outcomes. In addition, such politicians are also likely to be more successful in their efforts to foster development as the problems of smaller constituencies are likely to be more tractable than problems of larger constituencies.

Although having more legislators increases the free-rider problem, and increases the opportunity for politicians to claim credit for others’ activities, these dynamics are less likely in single-member district systems such as India’s where responsibilities are relatively clear [16]. Moreover, in parliamentary systems such as India, legislators from governing coalitions—rather than the opposition—might be particularly able to influence development outcomes, since the government is dependent on their votes for their majority [10].

To summarize, malapportionment or differences in relative representation might affect development as more politicians affect development to a greater degree, and as more politicians give enhance the ability of voters to hold politicians to account. More politicians could do the opposite—that is, they could make politicians less likely to deliver development due to collective action, free-riding and credit claiming problems—but these possibilities are less likely in India’s single member districts than would be the case in (say) a multimember district system [16].

## 3 Malapportionment in India’s states

As is the case in many countries, the Indian constitution secures for its citizens one-person one-vote. In the country’s state legislatures, which are the focus of this paper, this is ensured by providing for a universal franchise and decadal “delimitation” or redistricting. The country’s 29 states are divided into administrative districts, with relatively fixed boundaries. These administrative districts are divided into single-member assembly constituencies that elect Members of the Legislative Assemblies to state legislatures. During delimitation, each administrative district within a state is assigned single-member seats or constituencies in proportion to share of the state population. However, the Indian government froze redistricting in 1976, thereby causing an increasing degree of malapportionment across the country for the next several decades. The redistricting freeze expired in 2008, and the redistricting that followed has substantially equalized representation within state legislatures.

Following the literature, we may measure the degree of malapportionment across India’s districts using the relative representation index or RRI. The RRI is calculated as the seats per capita in a district divided by the seats per capita in the state ($v_{d,s,t}/{\bar{v}}_{s,t}$, where *v* is the seats per capita in the district, $\bar{v}$ is the seats per capita in the state, and *d*, *s*, and *t* denote the district, state and year, respectively). Within each state, the number of seats per capita would be a reasonable measure of representation. However, since our analysis uses data from India’s 15 largest states, we normalize this term by seats per capita in each state.

Due to data constraints, we calculate the RRI using the number of registered voters in each district, rather than population. More specifically, the Election Commission of India releases data on the number of registered voters, and not the population, of each district in election years. Reassuringly, population figures from the 2001 census are highly correlated with the number of registered voters in the election temporally closest to 2001 (*ρ* > 0.95). This is perhaps not surprising in India since the state rather than citizen is responsible for voter registration. Other works also use registered voters rather than population to measure malapportionment [1].

**Fig 1:**
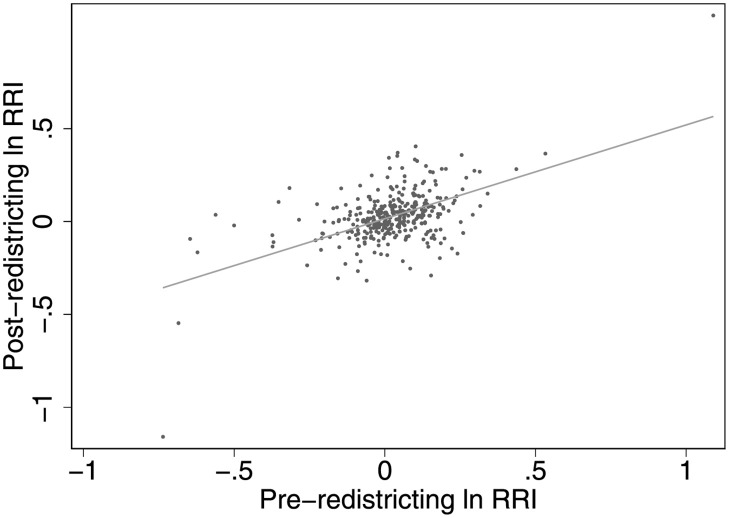
The relative representation index across India’s districts before and after redistricting. The line of best fit has slope 0.4.

Districts with RRIs greater than one are overrepresented, those with RRIs less than one are underrepresented, and districts whose RRI equals one give people a vote equal to the average. Fig 1 plots the log RRI before and after redistricting, illustrating the fact that the degree of malapportionment across India’s administrative districts was substantially reduced after redistricting (the slope of the fitted line is less than one).

Previous work has argued that the 1976 freeze in redistricting was implemented for apolitical and non-partisan reasons, to not reward high population growth states with additional representation [1]. Consistent with this argument, the freeze was instituted at a time of widespread paranoia about population growth in India, which also led to forced sterilizations. Furthermore, simulations suggest the freeze did not increase the seat shares of the Congress party that instituted it [1]. Lastly, the redistricting that did occur in May 2008 due to the lapsing redistricting freeze was implemented by a nonpartisan, constitutionally mandated commission and does not appear to have been influenced by partisanship [23].

**Fig 2:**
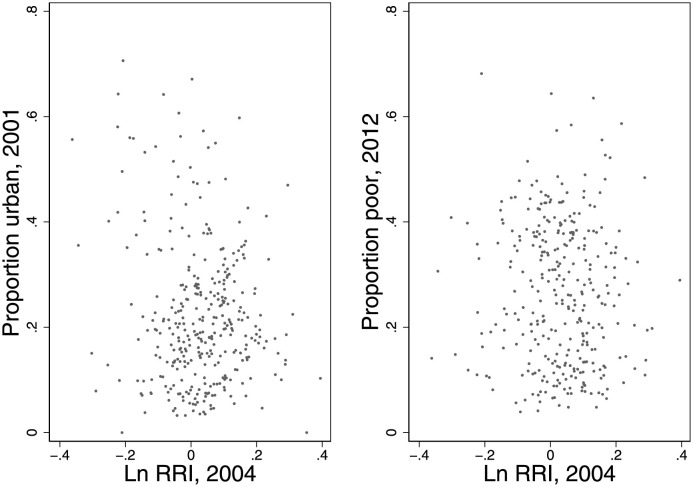
The relative representation index, urbanization and poverty.

Prior to redistricting, malapportionment in India underrepresented places with higher rates of birth or in-migration. This did not mean that underrepresented places were simply urban and poor, since districts in and around some large cities shrunk in relative terms even as they remained relatively rich. For example, close to 30 years of slow growth and in-migration resulted in Kolkata being overrepresented prior to delimitation. Its Relative Representation Index was 1.4 in 2006, when the last predelimitation election was held. Its RRI dropped to 0.9 in 2011, when the first post-delimitation election was held. To see this systematically, Fig 2 plots the bivariate relationships between the logarithm of the Relative Representation Index (constituencies with Ln RRI < 0 are underrepresented) and urbanization and poverty. The figure suggests that underrepresented places vary greatly in the degree to which they are urban and poor, thereby emphasizing their diversity.

## 4 Empirical strategy and data

To measure economic development at the subnational level, we employ median night light output for the villages in each of India’s administrative districts, as measured by the Defense Meteorological Satellite Program (DMSP) and made available at http://india.nightlights.io/. Using data mainly from Indonesia, it has been argued that Visible Infrared Imaging Radiometer Suite (VIIRS) nightlights data are superior to the DMSP data [24]. That said, a recent and India-specific validation exercise [25] finds that changes in DMSP nightlights data are highly correlated with district-level changes in some development indicators, namely non-farm, manufacturing and services employment (the elasticities of these variables with respect to nightlights range between 0.5–0.6). Furthermore, VIIRS data start in April 2012, whereas the DMSP data that we employ span 2004–2012.

Night lights have been increasingly used as a proxy for economic development [8–10], although a few works have also used it as a measure of urbanization [26] and/or electrification [20, 21]. We were able to find 43 scholarly works—working papers, published papers and books—that used night lights data. 61% of these used night lights to measure development, while another 20% used it to measure urbanization and/or population density. 16% of the works used nightlights to proxy for electrification and/or electricity consumption. Using a sample of developing countries, it is estimated that the elasticity of GDP growth with respect to light output growth is 0.3 [9]. In a robustness test, we also proxy for development using a count of the investment projects under implementation in India’s administrative districts.

Light output data have the advantage of being collected by satellite, objectively, and without the problems of survey non-response and political interference. They are therefore particularly useful in developing country contexts, and for measuring economic growth for subnational units for which GDP growth figures are unavailable. That said, nightlights data have problems as well, including sensor saturation or top-coding, light blooming beyond its origins, and technical difficulties due to cloudy weather. Since this is the case, we also analyze an second subnational measure of development that we introduce later. The median light output measure varies between 0–63.

The administrative district is the appropriate unit of analysis because it is the level at which most administrative decisions, including those relating to government-sponsored development programs, are made. We do not use electoral constituencies, which are nested among districts, as our unit of analysis since their boundaries change in 2008.

The key independent variable employed is the logarithm of the previously-introduced relative representation index (RRI) for each administrative district in India. To calculate the effect of representation on economic development, we estimate:

$$\begin{array}{c} Y_{d,s,t}=\alpha +\beta RRI_{d,s,t}+\gamma X_{d,s,t}+\delta_{s,t}+\eta_{d,s}+\epsilon_{d,s,t} \end{array}$$ (1)

where *Y* is log light output (in a robustness test, this is the log of the number of investment projects under implementation), *RRI* is the Relative Representation Index, *X* is a set of controls, including the lagged dependent variable and the logarithm of the number of registered voters, which could directly affect public goods, *δ*_*s*,*t*_ are state-year fixed effects, *η*_*d*,*s*_ are district fixed effects and *ϵ* is a normally distributed error term. *d* indexes administrative districts, which are nested within states (*s*), and *t* indexes time. Standard errors are clustered by state-year. The estimate of interest is *β*, the effect of changes in the RRI on changes in light output.

The data used in this paper are a balanced panel, covering India’s 15 largest states (with 88% of the country’s population) over 2004–2012. The data sources, construction and coverage details are noted in S1 Appendix and S1 Table; the data are summarized in S2 Table. Redistricting took effect in May 2008, and applied to all elections held from then on.

## 5 Malapportionment affects development

To start with, we use OLS to examine the correlation between log light output and the log RRI, controlling for the number of registered voters and lagged light output (regression 1 of Table 1). The controlled correlation is negative and statistically insignificant at conventional levels. This regression fails to control for the many confounds that state-year and district fixed effects would absorb. We include these in regression 2, and thereby implement Eq 1. This regression suggests that the estimate of the effect of changes in malapportionment due to redistricting on log light output is statistically and substantively significant. A 10% increase in representation causes a 0.6% increase in light output. Since the mean annual increase in light output is 2.5%, this change is equivalent to approximately 25% of annual light output growth.

**Table 1:** Does malapportionment affect light output?

	1	2	3
Ln Relative Representation Index (RRI)	-0.0230	0.0592**	0.102***
	(0.0171)	(0.0248)	(0.0299)
Ln RRI x Post-2008			-0.113***
			(0.0386)
Ln registered voters	0.0138***	0.0926***	0.0351
	(0.00391)	(0.0242)	(0.0290)
Lagged ln light output	0.964***	0.237***	0.223***
	(0.00678)	(0.0445)	(0.0441)
State-year fixed effects?	N	Y	Y
District fixed effects?	N	Y	Y
Observations	3222	3222	3222
Adjusted *R*-squared	0.87	0.96	0.96

*Notes*: The dependent variable is ln light output. Standard errors, clustered by state-year from regression 2 on, in parentheses.

* *p*< 0.10,

** *p*< 0.05,

*** *p*< 0.01.

A 10% change in malapportionment is relatively common in the sample, with 16% of district-years having a 10% or greater change in malapportionment. 19% of the district-years witnessed a change on nightlights of 6% or more. Our results are consistent both with reapportionment redistributing development across districts and also with it increasing overall levels of development. Ascertaining which of these is the case would require a different (state- rather than district-level) research design. Since the elasticities of electricity consumption and GDP growth with respect to light output are estimated to be 0.5 and 0.3 respectively [9], this suggests an electricity consumption increase of 0.3% and a GDP growth rate increase of 0.2%.

Having estimated the effect of representation on light output, we isolate the effects of redistricting on the effects of the RRI. To do so, we add the interaction of the RRI with a dummy set to one for the years 2008 and after (see regression 3; the uninteracted effect of the post-delimitation dummy is absorbed by the state-year fixed effects). The coefficient on the interaction term is negative and statistically significant. This suggests that the elasticity of night lights with respect to malapportionment is significantly different before and after redistricting, and further that reapportionment blunts the effects of malapportionment to a statistically significant degree. After delimitation, malapportionment—which has been drastically reduced—no longer influences nightlights.

**Fig 3:**
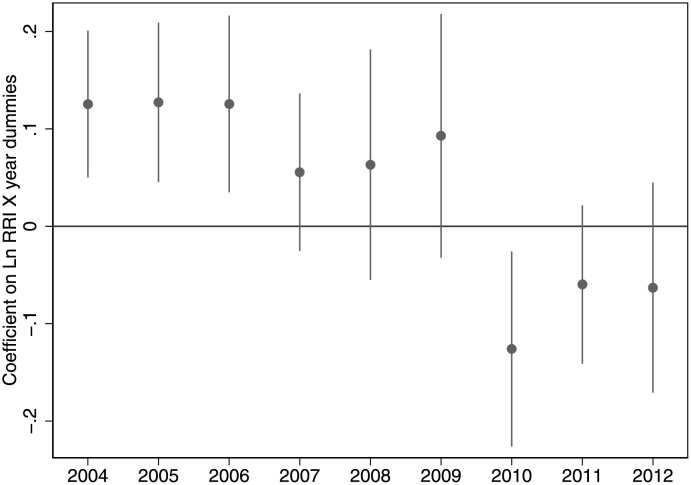
The effects of malapportionment over time. The figure plots the coefficients of an implementation of Eq 1, with Ln RRI interacted with annual dummies. Reapportionment occured in 2008.

To see how the effects of malapportionment evolve over time we interact the treatment (Ln RRI) with annual dummies, and plot these coefficients in Fig 3. Note that large standard errors might be partly driven by the fact that we have only 358 observations for each year. Consistent with the results in regression 3 of Table 1, the figure suggests that overrepresentation boosts nightlights in three of four years prior to the announcement of the new district boundaries in 2008, and does not thereafter. Draft reapportionment orders were gradually issued over 2005–2007, and so the attenuated effect of malapportionment in 2007 could be due to the anticipatory effects of the impending reapportionment.

A related issue is whether the effects of redistricting obtain after the new apportionment was announced in 2008 or after elections with the new boundaries were held between 2008 and 2012. To adjudicate between these possibilities, we interact the treatment (Ln RRI) with a dummy for the years 2008 and after in regression 1 of S3 Table (this is the same as regression 3 of Table 1 in the main text), and the treatment with a dummy that is set to one once elections with new boundaries are held in regression 2. The regressions suggest that the effects of malapportionment on development were blunted once redistricting was announced in 2008 (regression 1), rather than once elections with the new boundaries were held in 2008–2012 (regression 2). We do not emphasize the difference between these specifications since although the data for regression 1 is balanced in the sense that there is 4 years of pre-treatment and 5 years of post-treatment data for all states, the data for regression 2 is not balanced. Since state elections with the new constituency boundaries were held after the 5-year terms of each state legislature ended, we have 5 years of post-treatment data for Karnataka, Madhya Pradesh and Rajasthan, 4 years of post-treatment data for Andhra Pradesh, Haryana and Maharashtra, 3 years of post-treatment data for Bihar, 2 years of post-treatment data for Kerala, Tamil and West Bengal, and 1 year of post-treatment data for Gujarat, Himachal Pradesh, Punjab and Uttar Pradesh. In other words, the lack of a statistically significantly different effect of malapportionment on nightlights after redistricting in regression 2 might be driven by the fact that the samples before and after redistricting are different.

Our statistical analysis suggests that improved representation boosts economic development, as measured by night lights. To buttress our interpretation of the night lights data as development, we respecify our dependent variable as the (log) number of investment projects under implementation in India’s districts. The underlying data are from the Center for the Monitoring of the Indian Economy’s CapEx database, which tracks investments in infrastructure, manufacturing and services, implemented by the public and private sectors. Regression 1 of S4 Table examines the bivariate relationship between log projects and log RRI, and suggests no statistically significant relationship. Regression 2 implements our preferred specification with fixed effects, and suggests that a 10% increase in representation increases the number of projects by 1.9%, although this estimate is only statistically significant at 11%. Regression 3 adds the interaction of the RRI with a dummy for the years 2008 and after and suggests again that the effect of malapportionment (which is now estimated to be larger and statistically significant) is blunted after redistricting.

The main results are also robust to a number of additional tests, including to respecifying the dependent variable as the proportionate change in light output (regression 1 of S5 Table), to respecifying the independent variable as log seats, which is arguably more easily interpretable than the RRI (regression 2), to dropping the lagged value of log light output (concerns of Nickell bias might warrant dropping the term; regression 3), and to controlling for the (post-treatment) proportion of representatives that are members of governing coalitions (regression 4). We do not control for this variable in the main specification (or indeed others like it, such as partisan vote shares, the proportion of representatives in the cabinet, or spending on the Rajiv Gandhi Grameen Vidyutikaran Yojana electrification program) since its values are realized after the treatment is assigned. Controlling for post-treatment variables would bias the estimated effect of malapportionment, since malapportionment might work precisely through these variables [27, 28]. For an example of such a mechanism, please see the next section.

### 5.1 … particularly if legislators and voters are powerful

**Fig 4:**
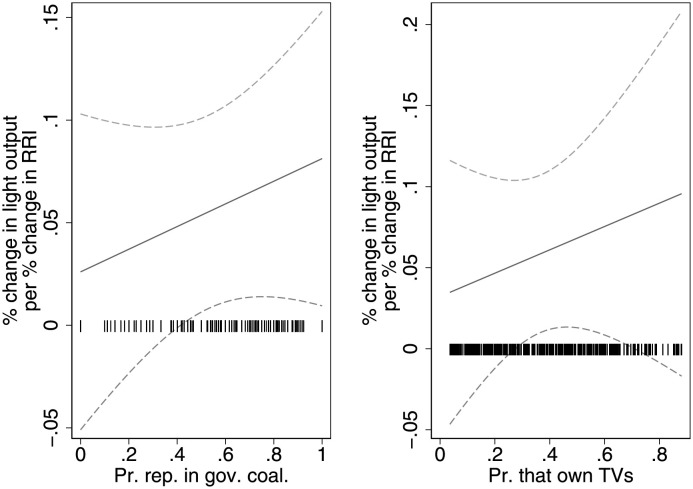
The effect of malapportionment on light output as the proportion of representatives in the governing coalition and those that own TV vary, with 95% confidence intervals. The coefficients underlying the plots are from the two regressions in S6 Table. The rug plots display the distribution of proportion of representatives in the governing coalition, and the proportion of the rural population that own TVs. See text for details.

As argued previously, malapportionment might affect development outcomes for top-down, strategic reasons, as politicians work to develop their constituencies. An observable implication of this mechanism is that the effects of additional representation should be particularly discernible in districts with powerful legislators. To test this possibility, we interact the proportion of state legislators in governing coalitions with the RRI (regression 1 of S6 Table). Note this analysis does not purport to be causal. We are merely examining whether the effects of the RRI that we have established are concentrated in the districts suggested by the theory. The interaction term is positive and statistically significant, suggesting that malapportionment does indeed boost light output largely in districts with a high proportion of representatives in governing coalitions. The first plot in Fig 4 maps the variation in the effect of representation on light output as the proportion of state legislators in governing coalitions varies. It suggests that the RRI boosts light output in the approximately 60% of district-years that have more than half of their state legislators in governing coalitions. This is consistent with the theory: while malapportionment affects development outcomes, it particularly does so when state legislators are in governing coalitions.

The second mechanism that we test is that representation affects light output bottom-up, as voters hold politicians to account. Since districts with high TV ownership rates should be particularly able to hold politicians to account (TV could embolden citizens, by making them aware of, or able to, demand their rights, and by improving their access to information), we should expect the positive effects of increased representation to particularly hold in these contexts. To test this, we interact the TV ownership rates (unfortunately, these data are only available from 2007/08 and are therefore not predetermined for the entire panel; regression 2 of S6 Table) with the log RRI. The interaction term is positive but is not statistically significant at conventional levels. Marginal effects are plotted in the second plot of Fig 4, which suggests that greater representation increases light output in most of the districts where at least 30% of the population own TVs. In regression 3 of S6 Table, we control for the two mechanisms simultaneously. Doing so substantially weakens the evidence in favor of all the mechanisms, possibly due to collinearity.

## 6 Conclusion

In this paper, we argued that malapportionment might affect economic development. Using data from India, we demonstrated that this is indeed the case. Before reapportionment, greater representation increases light output. Reapportionment blunts these effects. Weaker evidence suggests that the positive effect of greater representation is concentrated in constituencies with influential legislators and electorates. Malapportionment is ubiquitous and is, to an extent, unavoidable. We now know that in one important context, it has significant welfare consequences.

## Supporting information

S1 Appendix Data sources and construction. (PDF)

S1 Table Data coverage. (PDF)

S2 Table Summary statistics. (PDF)

S3 Table Alternative definitions of POST. (PDF)

S4 Table Robustness tests, 1 of 2. (PDF)

S5 Table Robustness tests, 2 of 2. (PDF)

S6 Table Mechanisms. (PDF)
