# Supplementary material for: The effects of malapportionment on economic development
Source: PLoS One. 2021 Dec 1;16(12):e0259150. doi: 10.1371/journal.pone.0259150 (PMC8635358; doi:10.1371/journal.pone.0259150)
Supplement: S1 Appendix — (PDF) [file pone.0259150.s001.pdf]

S1 Appendix: Data sources and construction

- Light output: Light output data for the median village in each district-month-year were downloaded from http://india.nightlights.io/ in February 2016. Most district-month-years had multiple measures of median light output, taken by different satellites. The median light output for each district-year was calculated in two steps. First, for each district-month-year, light output was set to the median light output as measured across multiple satellites. Second, for each district-year, light output was set to the median of the 12 monthly light output measures for that year.

- Number of projects under implementation: From the Center for Monitoring Indian Economy (CMIE).

- Ln registered voters: Linearly imputed using data on the registered number of voters in each district in election years. Underlying data are from Bhavnani, Rikhil R., 2014, "India National and State Election Dataset," doi:10.7910/DVN/26526, Harvard Dataverse, V2.

- Ln Relative Representation Index (RRI): Calculated using data from Bhavnani (2014) and registered voters data, calculated as above.

- Prop. of representatives in the governing coalition: Imputed from cabinet data. Cabinet data are coded using "Who's Who" directories, state government websites and responses to Right to Information requests.

- Prop. that own TVs: From the 2007/08 District Level Household and Facility Survey.
