# Supplementary material for: The effects of malapportionment on economic development
Source: PLoS One. 2021 Dec 1;16(12):e0259150. doi: 10.1371/journal.pone.0259150 (PMC8635358; doi:10.1371/journal.pone.0259150)
Supplement: S1 Table — (PDF) [file pone.0259150.s002.pdf]

S1 Table: Data coverage

State	Number of districts
Andhra Pradesh	21
Bihar	36
Gujarat	21
Haryana	19
Himachal Pradesh	12
Karnataka	25
Kerala	14
Madhya Pradesh	45
Maharashtra	29
Punjab	14
Rajasthan	26
Tamil Nadu	23
Uttar Pradesh	61
West Bengal	12
Total districts	358
Total years (2004–2012)	9
Sample size	3,222
