# Supplementary material for: The effects of malapportionment on economic development
Source: PLoS One. 2021 Dec 1;16(12):e0259150. doi: 10.1371/journal.pone.0259150 (PMC8635358; doi:10.1371/journal.pone.0259150)
Supplement: S2 Table — (PDF) [file pone.0259150.s003.pdf]

S2 Table: Summary statistics

	Mean	Std. Dev.	Min.	Max.
Ln light output	1.30	0.40	0.49	3.30
Prop. change in light output	0.02	0.15	-0.38	0.89
Ln Relative Representation Index (RRI)	0.03	0.16	-1.55	1.11
Ln registered voters	13.97	0.71	10.01	16.71
Prop. of representatives in the governing coalition	0.65	0.27	0.00	1.00
Prop. that own TVs	0.36	0.22	0.04	0.88
