# Supplementary material for: The effects of malapportionment on economic development
Source: PLoS One. 2021 Dec 1;16(12):e0259150. doi: 10.1371/journal.pone.0259150 (PMC8635358; doi:10.1371/journal.pone.0259150)
Supplement: S3 Table — (PDF) [file pone.0259150.s004.pdf]

S3 Table: Alternative definitions of POST

	1	2
Ln Relative Representation Index (RRI)	0.102*** (0.0299)	0.0682** (0.0274)
Ln RRI x Post-2008	-0.113*** (0.0386)	
Ln RRI x Post-redistricting		-0.0550 (0.0418)
Ln registered voters	0.0351 (0.0290)	0.0603** (0.0297)
Lagged ln light output	0.223*** (0.0441)	0.234*** (0.0448)
State-year fixed effects?	Y	Y
District fixed effects?	Y	Y
Observations	3222	3222
Adjusted R-squared	0.96	0.96

Notes: The dependent variable is ln light output. Standard errors are clustered by state-year and are in parentheses. * $p < 0.10$, ** $p < 0.05$, *** $p < 0.01$.
