# Supplementary material for: The effects of malapportionment on economic development
Source: PLoS One. 2021 Dec 1;16(12):e0259150. doi: 10.1371/journal.pone.0259150 (PMC8635358; doi:10.1371/journal.pone.0259150)
Supplement: S4 Table — (PDF) [file pone.0259150.s005.pdf]

S4 Table: Robustness tests, 1/2

| | 1 | 2 | 3 |
|---|---|---|---|
| Ln Relative Representation Index (RRI) | -0.108<br>(0.0890) | 0.223<br>(0.139) | 0.352**<br>(0.152) |
| Ln RRI x Post-2008 | | | -0.339**<br>(0.151) |
| Ln registered voters | 0.0633***<br>(0.0221) | 0.294**<br>(0.125) | 0.106<br>(0.143) |
| Lagged ln projects under implementation | 0.878***<br>(0.0110) | 0.202***<br>(0.0319) | 0.200***<br>(0.0317) |
| State-year fixed effects? | N | Y | Y |
| District fixed effects? | N | Y | Y |
| Observations | 2805 | 2805 | 2805 |
| Adjusted $R$-squared | 0.73 | 0.87 | 0.87 |

*Notes*: The dependent variable is the log of the number of investment projects under implementation. Standard errors, clustered by state-year from regression 2 on, in parentheses. * $p < 0.10$, ** $p < 0.05$, *** $p < 0.01$.
