# Supplementary material for: The effects of malapportionment on economic development
Source: PLoS One. 2021 Dec 1;16(12):e0259150. doi: 10.1371/journal.pone.0259150 (PMC8635358; doi:10.1371/journal.pone.0259150)
Supplement: S5 Table — (PDF) [file pone.0259150.s006.pdf]

S5 Table: Robustness tests, 2/2

Dependent variable:	Prop. change in light output	Ln light output		
	1	2	3	4
Ln Relative Representation Index (RRI)	0.0592**		0.0777***	0.0601**
	(0.0248)		(0.0260)	(0.0248)
Ln registered voters	0.0926***	0.0334	0.101***	0.0946***
	(0.0242)	(0.0249)	(0.0273)	(0.0238)
Lagged ln light output	-0.763***	0.237***		0.236***
	(0.0445)	(0.0445)		(0.0447)
Ln seats		0.0592**		
		(0.0248)		
Prop. of representatives in the governing coalition				0.0246**
				(0.0112)
State-year fixed effects?	Y	Y	Y	Y
District fixed effects?	Y	Y	Y	Y
Observations	3222	3222	3293	3222
Adjusted *R*-squared	0.73	0.96	0.96	0.96

Notes: Standard errors, clustered by state-year, in parentheses. * $p < 0.10$, ** $p < 0.05$, *** $p < 0.01$.
