# Supplementary material for: The effects of malapportionment on economic development
Source: PLoS One. 2021 Dec 1;16(12):e0259150. doi: 10.1371/journal.pone.0259150 (PMC8635358; doi:10.1371/journal.pone.0259150)
Supplement: S6 Table — (PDF) [file pone.0259150.s007.pdf]

S6 Table: Mechanisms

	1	2	3
Ln Relative Representation Index (RRI)	0.0260	0.0322	-0.0105
	(0.0393)	(0.0445)	(0.0626)
Ln RRI x Prop. of representatives from the governing coalition	0.0552		0.0606
	(0.0587)		(0.0599)
Prop. of representatives in the governing coalition	0.0225**		0.0227**
	(0.0112)		(0.0112)
Ln RRI x Prop. that own TVs		0.0720	0.0887
		(0.101)	(0.103)
Ln registered voters	0.0930***	0.0947***	0.0955***
	(0.0240)	(0.0248)	(0.0246)
Lagged ln light output	0.236***	0.236***	0.236***
	(0.0446)	(0.0445)	(0.0445)
State-year fixed effects?	Y	Y	Y
District fixed effects?	Y	Y	Y
Observations	3222	3222	3222
Adjusted R-squared	0.96	0.96	0.96

Notes: The dependent variable is ln light output. In regression 3–5, the uninteracted effects of literacy and TV ownership are absorbed by the district fixed effects. Standard errors, clustered by state-year, in parentheses. * $p < 0.10$, ** $p < 0.05$, *** $p < 0.01$.
